# Long-term exposure to high-altitude hypoxic environments reduces blood pressure by inhibiting the renin-angiotensin system in rats

**DOI:** 10.3389/fphys.2025.1565147

**Published:** 2025-04-15

**Authors:** Delong Duo, Junbo Zhu, Mengyue Wang, Xuejun Wang, Ning Qu, Xiangyang Li

**Affiliations:** ^1^ Research Center for High-Altitude Medicine, Qinghai University Medical College, Xining, China; ^2^ Qinghai Red Cross Hospital, Xining, China; ^3^ Qinghai Hospital of Traditional Chinese Medicine, Xining, China; ^4^ State Key Laboratory of Plateau Ecology and Agriculture, Qinghai University, Xining, China

**Keywords:** blood pressure, high-altitude hypoxia, renin-angiotensin system, spontaneously hypertensive rats, wistar kyoto rats

## Abstract

**Introduction:**

This study assesses the effects of chronic high-altitude hypoxia on blood pressure regulation in spontaneously hypertensive rats (SHR) and normotensive Wistar-Kyoto (WKY) rats, focusing on cardiovascular remodelling, hemodynamic alterations, and renin-angiotensin system (RAS) modulation.

**Methods:**

Eight-week-old male SHR and WKY rats were divided into four groups: the SHR high-altitude hypoxia group (SHR-H), WKY high-altitude hypoxia group (WKY-H), SHR control group (SHR-C), and WKY control group (WKY-C). The hypoxia groups were exposed to 4,300 m (PaO_2_: 12.5 kPa) for 10 weeks. Blood pressure was measured via non-invasive tail-cuff method, cardiac function via echocardiography, and right heart pressures via catheterization. Histopathological analysis included haematoxylin and eosin and Masson/Weigert staining for organ damage and vascular remodelling, whereas RAS components were assessed using immunohistochemistry.

**Results:**

The results showed that chronic hypoxia significantly reduced systolic blood pressure, diastolic blood pressure, and mean arterial pressure in SHR-H rats, but not in WKY-H rats. SHR-H rats showed a reduced ejection fraction, fractional shortening, systolic left ventricular anterior wall thickness, and diastolic left ventricular anterior wall thickness, increased left ventricular diastolic diameter, and left ventricular systolic diameter, whereas WKY-H showed only ejection fraction and fractional shortening decline. Both groups developed elevated mean pulmonary arterial pressure, right ventricular systolic pressure, and right ventricular end-diastolic pressure. SHR-H rats displayed aortic medial thinning, elastic fibre degradation, increased blood viscosity, and multi-organ damage (myocardial necrosis, pulmonary fibrosis), whereas WKY-H rats showed medial thinning and erythrocyte hyperplasia without fibrosis. Immunohistochemistry revealed suppression of the angiotensin-converting enzyme (ACE)-angiotensin II (Ang II)-angiotensin II type I (AT1) axis in SHR-H, whereas WKY-H exhibited reduced Ang I/II without ACE2 and Mas receptor (MasR) changes.

**Conclusion:**

Long-term hypoxic exposure at high-altitude reduces blood pressure in SHR rats, which may be attributed to a combination of cardiac functional compensation failure, vascular remodelling, and simultaneous inhibition of the ACE-Ang II-AT1R and ACE2-Ang1-7-MasR axes.

## 1 Introduction

More than 60 million people live in high-altitude areas (>2,500 m), 14.4 million of whom reside permanently at altitudes >3,500 m (Tremblay and Ainslie, 2021). In addition, millions of individuals from low-altitude regions travel to high-altitude areas for leisure, economic, and military purposes (Hui et al., 2016). High-altitude environments are characterized by hypoxia, low temperatures, aridity, and intense ultraviolet radiation. These conditions can cause systemic alterations, leading to various physiological or pathological changes, that influence the prevalence and severity of diseases, such as acute mountain sickness, polycythaemia, and pulmonary arterial hypertension (Penaloza and Arias-Stella, 2007; Narvaez-Guerra et al., 2018). The cardiovascular system consumes significant amounts of oxygen and energy and is highly susceptible to high-altitude hypoxia. When exposed to short- or long-term hypoxic conditions at high altitudes, the cardiovascular system undergoes physiological or pathological changes to ensure the delivery and supply of oxygen when arterial blood oxygen saturation is reduced (Narvaez-Guerra et al., 2018; Williams et al., 2022).

Hypertension is a primary risk factor for cardiovascular diseases, such as stroke, myocardial infarction, and heart failure, contributing significantly to the global disease burden (Mingji et al., 2015), with the World Health Organisation recognising hypertension as a global public health challenge (Zhang et al., 2022). Primary hypertension results from a complex interplay between genetic factors, age, and unhealthy lifestyle choices, and accounts for approximately 95% of all cases of hypertension. Although the development of primary hypertension has been researched, the exact causes and pathophysiological mechanisms remain unclear (Joint Committee for Guideline Revision, 2019). Furthermore, high-altitude hypoxic environments negatively affect blood pressure; patients with hypertension living at lower altitudes experience a further increase in blood pressure when exposed to a high-altitude hypoxic environment, and the extent of this increase is more pronounced than in individuals with normal blood pressure (Wu et al., 2007; Bilo et al., 2015). Research in the Tibetan region has shown a positive correlation between the prevalence of hypertension and altitude; for every 100 m increase in altitude, the prevalence of hypertension increases 2%, and for every 1,000 m increase, systolic blood pressure (SBP) rises by 15.6 mmHg (Mingji et al., 2015; Aryal et al., 2019). Thus, high-altitude-induced hypoxia is a risk factor for developing hypertension (Parati et al., 2018). Conversely, animal studies suggest that exposure to high-altitude hypoxic environments can reduce SBP in spontaneously hypertensive rats (SHR) by affecting thyroid hormone levels and metabolic changes (Henley and Tucker, 1987). To investigate these paradoxical effects, we employed the Wistar Kyoto (WKY) and SHR rat strains, which comprise a genetically matched model pair widely used in hypertension research (Pinto et al., 1998). The marked phenotypic divergence between these strains, coupled with their closely related genetic background, enables precise dissection of hypertension-specific pathophysiological alterations. This model system is particularly suited for studying hypoxia-induced blood pressure modulation.

Previous studies partially revealed the mechanisms by which high-altitude hypoxic exposure affects blood pressure (Duo et al., 2023). Antihypertensive medications such as calcium channel blockers and β-blockers show potential in reducing hypoxia-induced increases in blood pressure; however, current evidence does not support universally adjusting antihypertensive therapy for all hypertensive patients. Instead, close monitoring and contingency plans are recommended for patients with poorly controlled or labile hypertension (Luks, 2009). Further studies on the regulatory mechanisms of chronic high-altitude-induced hypoxia and blood pressure will aid in the management of associated diseases. This study aimed to determine the effects of chronic high-altitude exposure on blood pressure in hypertensive rats and the underlying mechanisms.

## 2 Results

### 2.1 Effect of high-altitude hypoxic conditions on blood pressure

**FIGURE 1:**
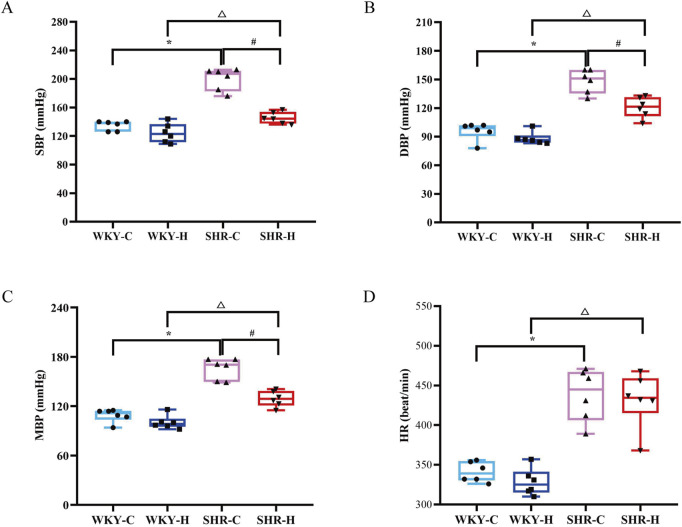
Effect of high-altitude hypoxic conditions on blood pressure in spontaneously hypertensive rats (SHRs) and Wistar Kyoto (WKY) rats. **(A)** Systolic blood pressure (SBP). **(B)** Diastolic blood pressure (DBP). **(C)** Mean blood pressure (MBP). **(D)** Heart rate (HR). Data are presented as the median (interquartile range) (n = 6). Note: WKY-C, SHR-C, WKY-H, and SHR-H refer to the WKY control group, SHR control group, WKY high-altitude hypoxia group, and SHR high-altitude hypoxia group, respectively. *P < 0.05 (vs. WKY-C), ^#^P < 0.05 (vs. SHR-C), and ^△^P < 0.05 (vs. WKY-H).

Compared with WKY control group (WKY-C) rats, SHR control group (SHR-C) rats exhibited increases in the SBP, diastolic blood pressure (DBP), mean blood pressure (MBP), and heart rate (HR) (*P <* 0.05). After 10 weeks of exposure to high-altitude hypoxic conditions, the SBP, DBP, and MBP of SHR high-altitude hypoxia group (SHR-H) rats decreased compared to SHR-C rats (*P <* 0.05). In contrast to WKY-H rats, SHR-H rats showed increased levels of SBP, DBP, MBP, and HR (*P <* 0.05) (Figure 1).

**FIGURE 2:**
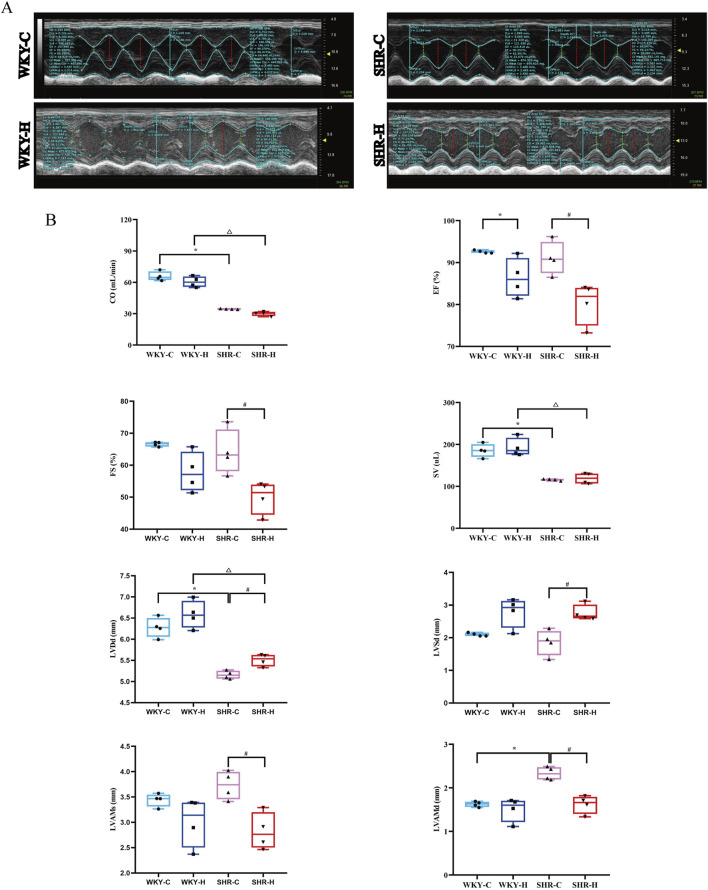
Effect of high-altitude hypoxic conditions on cardiac output (CO), ejection fraction (EF), fractional shortening (FS), stroke volume (SV), left ventricular end-diastolic diameter (LVDd), left ventricular internal dimension systole (LVSd), left ventricular end-systolic anterior wall thickness (LVAMs), and left ventricular end-diastolic anterior wall thickness (LVAMd) in spontaneously hypertensive rats (SHRs) and Wistar Kyoto (WKY) rats. **(A)** Echocardiography. **(B)** Left ventricular functional indices. Data are presented as the median (interquartile range) (n = 4). Note: WKY-C, SHR-C, WKY-H, and SHR-H refer to the WKY control group, SHR control group, WKY high-altitude hypoxia group, and SHR high-altitude hypoxia group, respectively. *P < 0.05 (vs. WKY-C), ^#^P < 0.05 (vs. SHR-C), and ^△^P < 0.05 (vs. WKY-H).

Echocardiography was then used to assess the effect of high-altitude hypoxic environments on cardiac function. Compared with WKY-C rats, SHR-C rats exhibited decreases in stroke volume and cardiac output (*P <* 0.05) and an increase in diastolic left ventricular anterior wall thickness (LVAMd) (*P <* 0.05). After 10 weeks of exposure to high-altitude hypoxic conditions, the ejection fraction (EF), systolic left ventricular anterior wall thickness (LVAMs), and LVAMd were decreased in SHR-H rats compared with in SHR-C rats (*P <* 0.05). Additionally, left ventricular diastolic diameter (LVDd) and left ventricular systolic diameter (LVSd) showed increases in SHR-H rats compared with in SHR-C rats (*P <* 0.05). Compared with WKY-C rats, WKY-H rats showed decreases in EF and FS (*P <* 0.05). Furthermore, compared with WKY-H rats, SHR-H rats showed decreases in CO, stroke volume, and LVDd (*P <* 0.05) (Figure 2). These results indicate that long-term exposure to high-altitude reduces blood pressure in SHR rats, whereas cardiac functional indices such as EF, FS, LVAMs, and LVAMd were all decreased. Concurrently, LVDd and LVSd were increased. In contrast, WKY rats showed no significant changes in blood pressure, but their EF and FS declined and LVSd increased. These findings indicate that hypoxic conditions exert less pronounced effects on WKY rats than on SHR rats.

### 2.2 Effects of high-altitude hypoxic conditions on haemodynamics in WKY rats and SHRs

**FIGURE 3:**
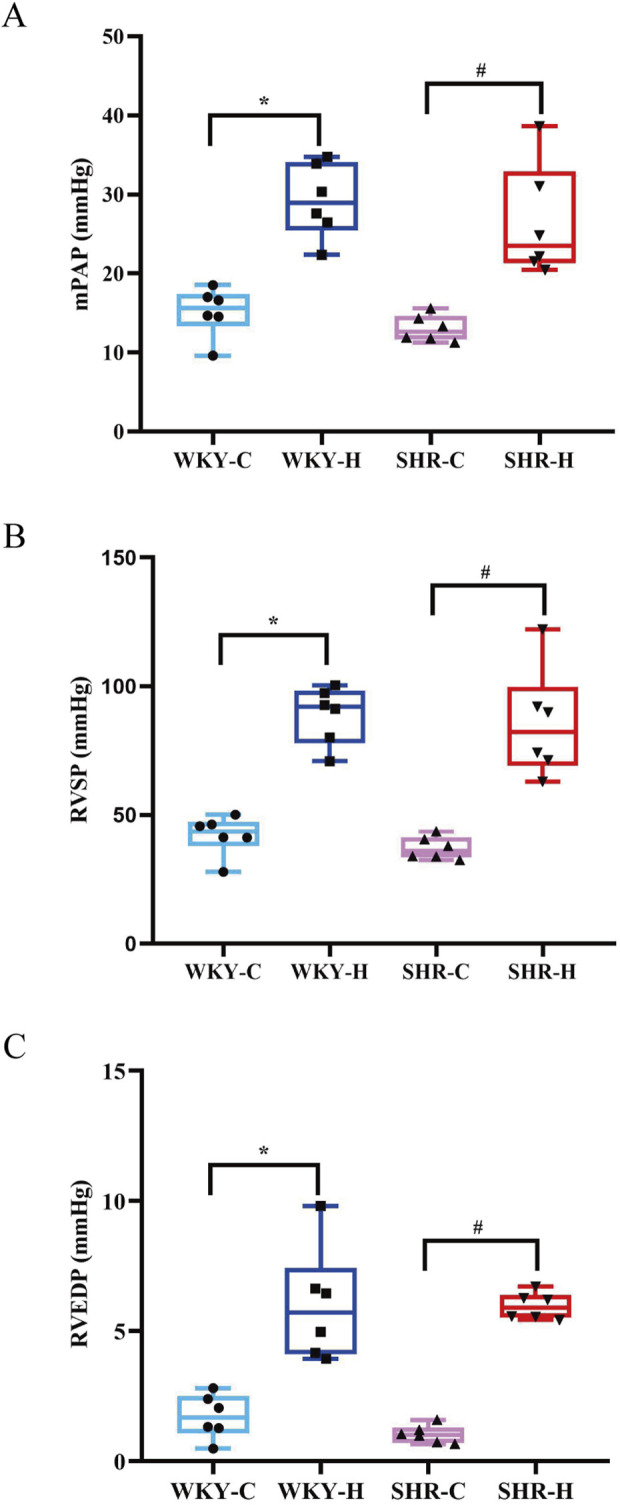
Effect of high-altitude hypoxic conditions on mean pulmonary arterial pressure (mPAP), right ventricular systolic pressure (RVSP), and right ventricular end-diastolic pressure (RVEDP) in Wistar Kyoto (WKY) rats and spontaneously hypertensive rats (SHRs). **(A)** mPAP, **(B)** RVSP, and **(C)** RVEDP. Data are presented as the median (interquartile range) (n = 6). Note: WKY-C, SHR-C, WKY-H, and SHR-H refer to the WKY control group, SHR control group, WKY high-altitude hypoxia group, and SHR high-altitude hypoxia group, respectively. *P < 0.05 (vs. WKY-C) and ^#^P < 0.05 (vs. SHR-C).

After 10 weeks of exposure to high-altitude hypoxic conditions, WKY-C rats exhibited increases in the mean pulmonary arterial pressure (mPAP), right ventricular systolic pressure (RVSP), and right ventricular end-diastolic pressure (RVEDP) compared with the values in WKY-H rats (*P <* 0.05). Similarly, SHR-H rats showed increase mPAP, RVSP, and RVEDP compared with those in SHR-C rats (*P <* 0.05) (Figure 3). These results indicate that long-term exposure high-altitude induces significant mPAP and right ventricular dysfunction in both WKY and SHR rats.

### 2.3 Effects of high-altitude hypoxic conditions on routine blood and biochemical parameters

**FIGURE 4:**
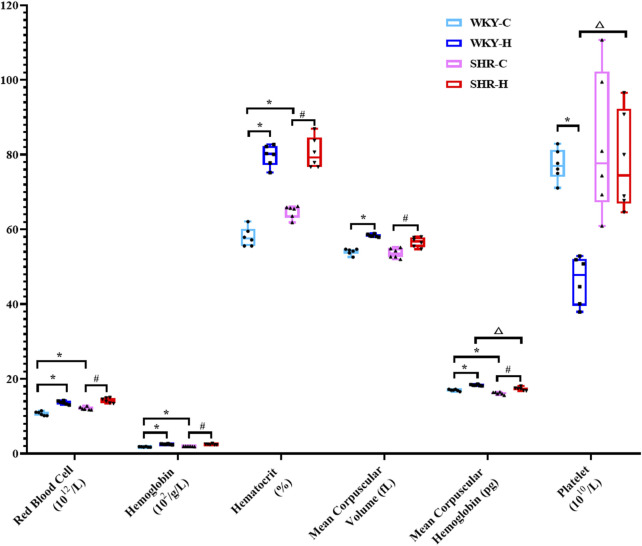
Changes in white blood cell, red blood cell, haemoglobin, haematocrit, mean corpuscular volume, mean corpuscular haemoglobin, and platelet levels in Wistar Kyoto (WKY) rats and spontaneously hypertensive rats (SHRs) after exposure to high-altitude hypoxic conditions. Data are presented as the median (interquartile range) (n = 6). Note: WKY-C, SHR-C, WKY-H, and SHR-H refer to the WKY control group, SHR control group, WKY high-altitude hypoxia group, and SHR high-altitude hypoxia group, respectively. *P < 0.05 (vs. WKY-C), ^#^P < 0.05 (vs. SHR-C), and ^△^P < 0.05 (vs. WKY-H).

The routine blood changes in SHRs and WKY rats were compared (Figure 4). Compared with WKY-C rats, SHR-C rats exhibited increases in the red blood cell (RBC) count, haemoglobin, and haematocrit concentrations (*P <* 0.05), along with a decrease in mean corpuscular haemoglobin (MCH) (*P <* 0.05). After 10 weeks of exposure to high-altitude hypoxic conditions, WKY-H rats showed increased RBC counts, haemoglobin, and haematocrit concentrations, mean corpuscular volume (MCV), and MCH levels compared to those in WKY-C rats (*P <* 0.05), while platelet levels decreased (*P <* 0.05). Similarly, compared with SHR-C rats, SHR-H rats exhibited increases in RBC count, haemoglobin, haematocrit concentrations, MCV, and MCH levels (*P <* 0.05). Furthermore, compared with WKY-H rats, SHR-H rats showed decreases in MCH and an increase in platelet levels (*P <* 0.05).

**FIGURE 5:**
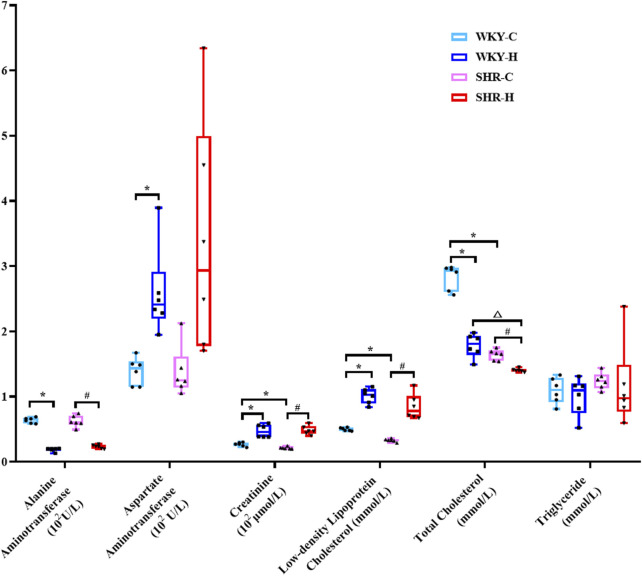
Changes in alanine aminotransferase, aspartate aminotransferase, creatinine, glucose, urea, low-density lipoprotein cholesterol, total cholesterol, and triglyceride levels in Wistar Kyoto (WKY) rats and spontaneously hypertensive rats (SHRs) after exposure to high-altitude hypoxic conditions. Data are presented as the median (interquartile range) (n = 6). Note: WKY-C, SHR-C, WKY-H, and SHR-H refer to the WKY control group, SHR control group, WKY high-altitude hypoxia group, and SHR high-altitude hypoxia group, respectively. *P < 0.05 (vs. WKY-C), #P < 0.05 (vs. SHR-C), and ^△^P < 0.05 (vs. WKY-H).

The biochemical parameters of SHRs and WKY rats were compared (Figure 5). Compared with WKY-C rats, SHR-C rats exhibited decreases in the creatinine, low-density lipoprotein cholesterol (LDL-C), and total cholesterol (TC) levels (*P <* 0.05). After 10 weeks of exposure to high-altitude hypoxic conditions, WKY-H rats showed increased aspartate aminotransferase, creatinine, and LDL-C levels compared to those in WKY-C rats (*P <* 0.05). Additionally, alanine aminotransferase and TC levels were decreased in WKY-H rats compared to those in WKY-C rats (*P <* 0.05). Compared with the SHR-C rats, SHR-H rats showed increases in creatinine and LDL-C levels (*P <* 0.05), along with decreases in alanine aminotransferase and TC levels (*P <* 0.05). Furthermore, compared with WKY-H rats, SHR-H rats showed decreases in TC levels (*P <* 0.05). These results indicate that long-term exposure to high altitude drives WKY rats to prioritise erythrocytic compensation for enhanced oxygen transport, albeit with accompanying hepatic injury, whereas SHR rats are more susceptible to renal function deterioration due to their hypertensive predisposition.

### 2.4 Effect of high-altitude hypoxic conditions on heart, liver, lung, and kidney tissue morphology in SHRs and WKY rats

**FIGURE 6:**
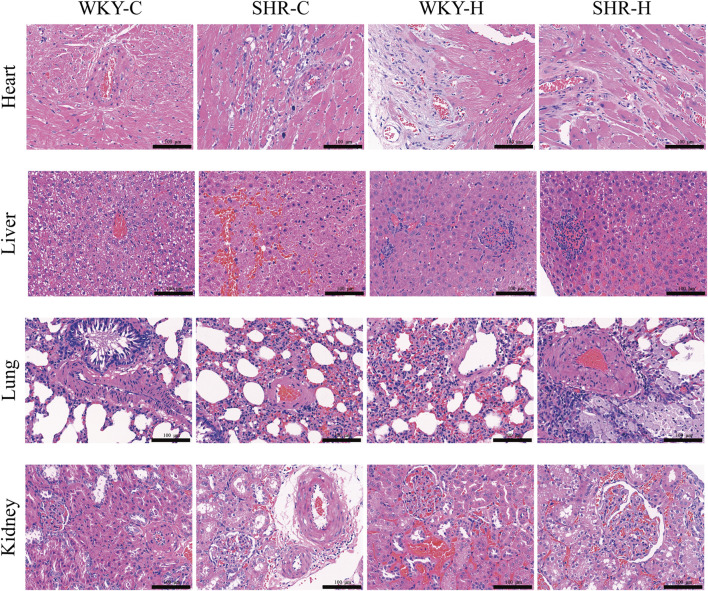
Effect of high-altitude hypoxic conditions on heart, liver, lung, and kidney tissues in spontaneously hypertensive rats (SHRs) and Wistar Kyoto (WKY) rats. Haematoxylin and eosin staining. Note: WKY-C, SHR-C, WKY-H, and SHR-H refer to the WKY control group, SHR control group, WKY high-altitude hypoxia group, and SHR high-altitude hypoxia group, respectively.

Haematoxylin and eosin staining indicated that the heart, liver, lungs, and kidneys of the WKY-C rats were intact (Figure 6). However, the SHR-C rats exhibited myocardial fibre degeneration and necrosis, accompanied by proliferation of interstitial fibrous tissue around necrotic areas and cytolysis. Additionally, focal hepatocyte necrosis was observed along with slight hyperplasia of the microstructure in the necrotic region, congestion of the hepatic sinusoids, and bleeding. The alveolar epithelial cells displayed degenerative necrosis, the pulmonary interstitium was congested, and the vascular walls were thickened. The renal interstitium was expanded and congested with a small number of infiltrating inflammatory cells. Following exposure to high-altitude hypoxic conditions, both the WKY-H and SHR-H rats exhibited myocardial fibre degeneration and necrosis, cytolysis, interstitial vessel congestion, lumen dilation, and accumulation of numerous RBCs (Figure 6). The liver cells showed focal necrosis with a slight increase in fibrous tissue in the affected areas. The hepatic sinusoids were dilated and congested, and congestion occurred in the portal area. The cells lining the air sacs in the lungs underwent degenerative necrosis with infiltration of numerous inflammatory cells. Capillaries in the outer layer of the kidney were dilated and congested, with minimal cell death. Simultaneously, the SHR-H rats exhibited pulmonary interstitial congestion, thickened vascular walls, and fibrous tissue proliferation around the alveolar septa and blood vessels. Additionally, slight mesangial proliferation in the kidneys and dilated and congested interstitial blood vessels were observed.

We employed a semi-quantitative four-point scoring system (0–4 points representing the normal range, minor, mild, moderate, and severe degrees of lesions) to assess the extent of lesions in the heart, liver, lungs, and kidney (Zhang and Yao, 2021). The median scores for myocardial fibre degeneration and necrosis in the WKY-C, SHR-C, WKY-H, and SHR-H groups were 0, 1, 1.5, and 2, respectively. The median scores for myocardial fibre proliferation were 0, 1, 1, and 1.5, respectively. The median scores for hepatic sinus congestion and haemorrhage were 0, 2, 1, and 1.5, respectively. The median scores for pulmonary vascular wall thickening were 0, 1, 0, and 1.5, respectively. The median scores for renal vascular congestion were 0, 1, 2, and 2, respectively. There were significant differences in the scores of all lesion degrees among the four groups (*P <* 0.05).

### 2.5 Effect of high-altitude hypoxic conditions on abdominal aortic morphology and collagen and elastic fibre content

**FIGURE 7:**
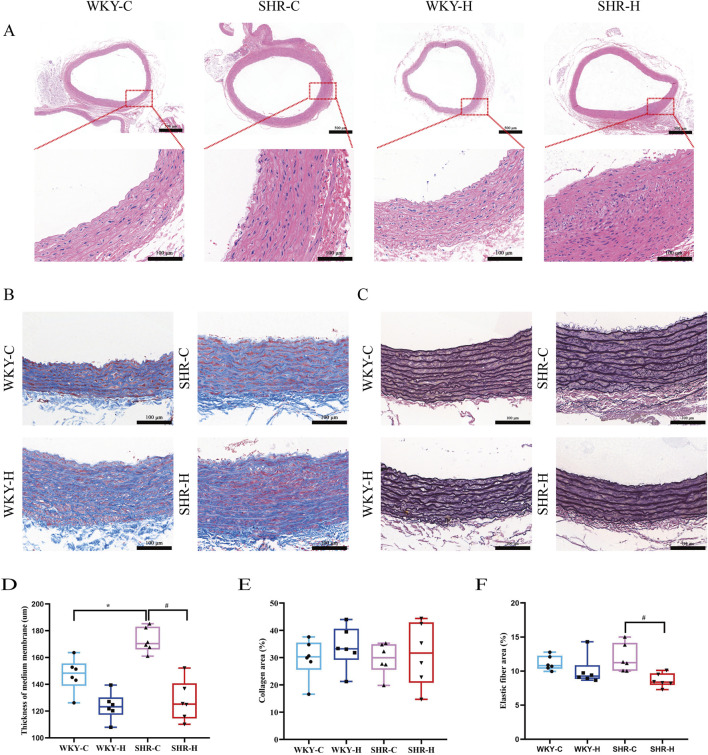
Effect of high-altitude hypoxic conditions on abdominal aortic morphology, collagen, and elastic fibres in spontaneously hypertensive rats (SHRs) and Wistar Kyoto (WKY) rats. **(A)** Haematoxylin and eosin staining for morphology. **(B)** Masson’s staining for collagen. **(C)** Weigert’s staining for elastic fibres. **(D)** Abdominal aortic medial thickness. **(E)** Collagen fibre area. **(F)** Elastic fibre area. Data are presented as the median (interquartile range) (n = 5). Note: WKY-C, SHR-C, WKY-H, and SHR-H refer to the WKY control group, SHR control group, WKY high-altitude hypoxia group, and SHR high-altitude hypoxia group, respectively. *P < 0.05 (vs. WKY-C) and ^#^P < 0.05 (vs. SHR-C).

Haematoxylin and eosin staining indicated that compared with those in the WKY-C rats, the local endothelial cells of the intima layer of the abdominal aortic tissues in the SHR-C rats were shed (Figure 7A). The elastic fibres in the middle membrane were broken and disordered, and the adventitial layer thickened. After 10 weeks of exposure to high-altitude hypoxic conditions, compared with those of the SHR-C rats, the local endothelial cells of the intimal layer of the SHR-H rats were shed, the elastic fibres in the medium membrane were broken and disordered, and local thickening and smooth muscle hyperplasia were observed. The inner membrane of the WKY-H rats was thinner than that of the WKY-C rats, and the endothelial cells were flat and close to the inner elastic fibres. The elastic fibres in the middle membrane were broken and disordered, and the outermost layer was thick. Furthermore, we used a semi-quantitative four-point scoring system (0–4 points representing the normal range, minor, mild, moderate, and severe degrees of lesions) to assess the extent of aortic lesions (Zhang and Yao, 2021). The median scores for endothelial cell shedding in the WKY-C, SHR-C, WKY-H, and SHR-H groups were 0, 0.5, 1, and 1, respectively, whereas the median scores for elastic fibre rupture were 0, 2, 2, and 2, respectively. There were significant differences in the scores for endothelial cell shedding and elastic fibre rupture among the four groups (*P <* 0.05).

Masson’s trichrome and Weigert staining were performed to detect the collagen and elastic fibre contents, respectively, in the abdominal aorta, with the relative content values of each expressed as area fractions. The elastic fibre content decreased in WKY-H and SHR-H rats (Figures 7B, C). Quantitative analysis showed that the thickness of the medial layer in SHR-C rats exhibited was increased compared with that in WKY-C rats (*P <* 0.05). After 10 weeks of exposure to high-altitude hypoxic conditions, compared with that in SHR-C rats, the thickness of the medial layer in SHR-H rats was reduced (*P* < 0.05, Figure 7D). The percentage of collagen fibres did not differ between groups (*P* > 0.05, Figure 7E). Simultaneously, the percentage of elastic fibre expression area in SHR-H rats was reduced compared with that in SHR-C rats (*P* < 0.05, Figure 7F). These results indicate that during long-term exposure to high-altitude hypoxia, SHR rats are more prone to muti-organ fibrosis and vascular elastic degradation because of their underlying hypertension.

### 2.6 Effects of high-altitude hypoxic conditions on angiotensin I (ang I), angiotensin-converting enzyme (ACE), ang II, AT1R, ACE2, ang 1–7, and mas receptor (MasR) expression

**FIGURE 8:**
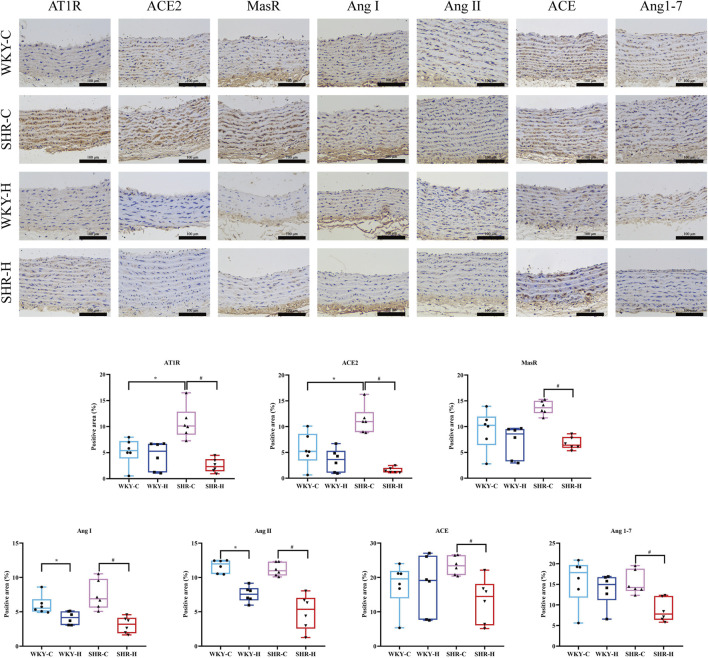
Effect of high-altitude hypoxic conditions on angiotensin Ⅰ (Ang I), angiotensin-converting enzyme (ACE), angiotensin II (Ang II), angiotensin II type I receptor (AT1R), angiotensin-converting enzyme 2 (ACE 2), angiotensin 1–7 (Ang [1–7]), and Mas receptor (MasR) expression in Wistar Kyoto (WKY) rats and spontaneously hypertensive rats (SHRs). Data are presented as the median (interquartile range) (n = 6). Note: WKY-C, SHR-C, WKY-H, and SHR-H refer to the WKY control group, SHR control group, WKY high-altitude hypoxia group, and SHR high-altitude hypoxia group, respectively. *P < 0.05 (vs. WKY-C) and ^#^P < 0.05 (vs. SHR-C).

Compared with those in WKY-C rats, immunohistochemistry revealed that the positive areas expressing AT1R and ACE2 were increased in SHR-C rats (*P* < 0.05). After 10 weeks of exposure to high-altitude hypoxic conditions, compared with those in WKY-C rats, the positive areas expressing Ang I and Ang II were reduced in WKY-H rats (*P <* 0.05); compared with those in SHR-C rats, the positive areas expressing Ang I, ACE, Ang II, AT1R, ACE2, Ang1-7, and MasR were decreased in SHR-H rats (*P <* 0.05). (Figure 8). These results indicate that during long-term high-altitude hypoxic exposure, the expression of Ang I and Ang II was reduced in WKY rats, whereas ACE2 and MasR levels remained unaffected. In contrast, SHR rats exhibited simultaneous suppression of both the ACE-Ang II-AT1R and ACE2-Ang1–7-MasR axes.

## 3 Discussion

This study described the effects of chronic high-altitude-induced hypoxia on the cardiovascular system, haematological indices, tissue morphology, and renin-angiotensin system (RAS) in WKY rats and SHRs. Chronic hypoxic exposure lowered SBP, DBP, and MBP in SHRs, accompanied by alterations in cardiac structure and contractile function, as evidenced by increases in mPAP, RVEDP, RVSP, LVDd, and LVSd, and decreases in EF, FS, LVAMs, and LVAMd. Moreover, high-altitude hypoxic exposure increased RBC count, haemoglobin and haematocrit concentration, and creatinine level, as well as MCV and MCH, and affected the heart, liver, lungs, and kidneys. Additionally, it reduced the thickness of the medial layer of the abdominal aorta, decreased the integrity of elastic fibres, and downregulated RAS activity, as evidenced by changes in the expression of the RAS factors Ang I, ACE, Ang II, AT1R, ACE2, Ang 1–7, and MasR. These findings suggest that the reduction in blood pressure in SHR rats induced by high-altitude hypoxic exposure is associated with damage to the cardiac structure and function, reduced integrity of elastic fibres in the vascular wall, and RAS activity inhibition.

Exposure to high-altitude hypoxic conditions causes various physiological and pathological changes in the body. The primary function of the cardiovascular system is to ensure that CO matches the metabolic demands of the body. Under hypoxic conditions, the cardiovascular system must be adjusted to ensure oxygen delivery when arterial blood oxygen saturation decreases (Champigneulle et al., 2024). Exposure to high-altitude hypoxic conditions leads to alveolar hypoxia and hypoxaemia, which induce pulmonary vasoconstriction, increase pulmonary vascular resistance, and elevate pulmonary artery pressure. This aggravates the afterload on the right ventricle, increasing the RVSP (Parati et al., 2018; Stembridge et al., 2021). With a persistent rise in pulmonary artery pressure exceeding the compensatory capacity of the right ventricle, the right ventricular output decreases and the end-diastolic volume in the right ventricle increases, prompting right ventricular hypertrophy, ultimately leading to right heart failure (Doutreleau et al., 2022). Simultaneously, high-altitude hypoxic conditions cause dysfunction in left ventricular contractile function, with a reduction in the EF, weakened myocardial contractility, and insufficient blood propulsion (Parati et al., 2018). Using animal echocardiography and jugular vein catheterisation to measure cardiac structure and function, we found that long-term high-altitude-induced hypoxia changes rat cardiopulmonary haemodynamics, leading to increased rat pulmonary artery and right ventricular systolic/diastolic pressure and left ventricular structural and contractile dysfunction, demonstrated by decreases in EF, FS, and thickness of the left ventricular anterior wall during both systole and diastole, whereas the LVDd and LVSd were increased. High-altitude-induced hypoxia also resulted in alveolar epithelial cell degeneration and necrosis, inflammatory cell infiltration, congested pulmonary interstitium, and proliferation of interstitial or perivascular fibrous tissues. It damaged myocardial cells, causing degeneration and necrosis of myocardial fibres, as well as proliferation of interstitial fibrous connective tissue. The combined evidence of haemodynamics (increased RVEDP) and histology (myocardial necrosis) suggests that the right ventricle is in a state of compensated critical condition. This finding is consistent with the increased afterload on the right ventricle due to pulmonary vasoconstriction and pulmonary hypertension caused by chronic hypoxia, which may ultimately lead to compensatory hypertrophy of the right ventricle (Vonk Noordegraaf et al., 2019). These cardiac alterations may be one reason for the observed reduction in blood pressure. Additionally, the impact of high-altitude-induced hypoxia on vascular resistance has been noted, as it can reduce the reactivity of SHR vessels to vasoconstrictors and lower vascular resistance (Henley and Tucker, 1986; Pijacka et al., 2018). This study showed that long-term high-altitude-induced hypoxia causes changes in the structure of the rat abdominal aorta. The reduction in the thickness of the medial layer of the abdominal aorta, degradation of collagen fibres, and decreases in the content and integrity of elastic fibres suggest that hypoxia-induced changes in vascular structure and function may be another reason for the decrease in blood pressure associated with high-altitude-induced hypoxia.

Haematological and serum biochemical indicators are important markers for assessing the health status of the body and have implications for the diagnosis and treatment of diseases. The production of erythropoietin by the kidneys stimulates the generation of RBCs, and the haemoglobin concentration further increases to compensate for the body’s oxygen needs when the arterial oxygen partial pressure decreases, which is consistent with our previously findings (Zhu et al., 2022). The current study demonstrated that RBCs, serving as oxygen carriers and sensors, are particularly sensitive to hypoxia. During exposure to high-altitude hypoxic conditions, the RBC count and haemoglobin level increased, which enhanced the oxygen-carrying capacity of RBCs to overcome the reduction in oxygen delivery to tissues and maintain the function and survival of all cells in the body. However, these haematological changes typically lead to an increase in blood viscosity and adverse effects on ventilation and acid-base balance. Increased blood viscosity is also accompanied by an increase in pulmonary artery pressure and peripheral vascular resistance, leading to increased cardiac effort, thus impacting cardiac function (Stembridge et al., 2021). In this study, both WKY rats and SHRs were maintained under the same experimental conditions and fed the same diet, which eliminated the influence of diet on haematological and biochemical indicators. The results indicate that high-altitude hypoxia induces erythrocytosis, which increases blood viscosity, which, along with pulmonary hypertension, exacerbates right ventricular afterload, leading to elevated right ventricular end-diastolic pressure.

Studies of hepatic and renal dysfunction and lipid metabolism abnormalities in rats under high-altitude hypoxic conditions have revealed intricate interactions between liver functional changes and the drug-metabolising enzyme system. Abnormal elevation of ALT and AST, as critical biomarkers of liver injury, is significantly associated with altered activity of drug-metabolising enzymes such as cytochrome (CYP450). CYP450 directly influences drug metabolic efficiency through its substrate specificity, induction, or inhibition states and is closely linked to the pathogenesis of drug-induced liver injury (Zhou et al., 2018; Yu et al., 2014). Furthermore, abnormal liver function may interfere with the conversion of thyroxine to its active form, which is consistent with the metabolic hypothesis proposed by Henley and Tucker (1987). Under high-altitude hypoxia, hepatocytes experience oxidative stress and energy metabolism dysfunction, leading to increased membrane permeability and subsequent release of ALT/AST into the bloodstream. Concurrently, hypoxia-induced alterations in the hepatic microenvironment trigger epigenetic modifications, which further influence the expression and catalytic activity of CYP450 isoforms (Singhal et al., 2014). From a lipid homeostasis perspective, high-altitude hypoxia may mediate the inverse regulation of LDL-C and TC through dual pathways. On one hand, high-altitude hypoxia enhances fat mobilisation and downregulates hepatic LDL receptor expression to reduce LDL-C clearance. On the other hand, high-altitude hypoxia inhibits cholesterol synthesis and promotes cholesterol conversion to bile acids and excretion, ultimately leading to a characteristic metabolic phenotype involving elevated LDL-C levels and decreased TC levels (Zhang et al., 2018).

The RAS is an important fluid-regulating system in the body, playing a crucial role in regulating blood pressure (Ma et al., 2023). Within the RAS, there are two mutually inhibitory functional axes: one is the classical ACE-Ang II-AT1R axis, and the other is the ACE2-Ang1–7-MasR axis. These two axes play different roles under physiological and pathological conditions. The classical ACE-Ang II-AT1R axis primarily promotes vasoconstriction, cell proliferation, and inflammatory responses, whereas the ACE2-Ang1–7-MasR axis has opposing effects, promoting vasodilation, anti-proliferation, and anti-inflammatory responses (Igić and Škrbić, 2014; Verand-Braga et al., 2020). In the systemic RAS, the blood pressure and electrolyte balance are regulated through the secretion of aldosterone from the adrenal cortex. Activation of the local RAS in the vascular wall leads to the proliferation and migration of vascular smooth muscle cells, resulting in vascular remodelling and fibrosis. This process not only increases vascular stiffness but may also promote the development of hypertension (De Mello and Frohlich, 2014; Sztechman et al., 2018). The impact of high-altitude hypoxia on the RAS has been extensively studied. Exposure to high-altitude hypoxic conditions suppresses renin activity and reduces Ang II and aldosterone (Revera et al., 2017). Parati et al. (2014) found that in healthy volunteers exposed to acute exposure to an altitude of 3,400 m for 3 days, the RAS was partially inhibited. The RAS was completely suppressed at 5,400 m and plasma renin activity, Ang Ⅱ concentration, and aldosterone levels were significantly reduced. Studies on patients with hypertension have also confirmed that exposure to high-altitude hypoxic conditions suppresses the RAS (Bilo et al., 2015). Moreover, long-term inhibition of the RAS can reduce vascular stiffness and alter the structure and function of blood vessels (Mitchell et al., 2007). Although studies in humans indicate that the RAS is suppressed under high-altitude hypoxia, these findings reflect human-specific pathophysiology and should not be directly equated with SHR rat models due to significant differences in genetic and adaptive responses. This study showed that during long-term high-altitude hypoxic exposure, WKY rats selectively suppressed overactivation of the ACE-Ang II-AT1R axis while preserving the integrity of the ACE2-Ang1–7-MasR axis. In contrast, SHR rats exhibited simultaneous suppression of both the ACE-Ang II-AT1R and ACE2-Ang1–7-MasR axes. This finding suggesting that long-term hypoxia leads to sustained decreases in vascular tone by inhibiting RAS compensatory mechanisms. Additionally, this study provides the first evidence that normotensive individuals achieve high-altitude adaptation through ACE2-mediated selective inhibition of the RAS axis, whereas hypertensive models develop systemic RAS dysfunction due to genetic defects causing the collapse of the ACE/ACE2 regulatory network.

Notably, our findings in SHRs contrast with human epidemiological data showing an increased prevalence of hypertension at high altitudes. This discrepancy may stem from fundamentally different physiological regulatory mechanisms, leading to increased blood pressure in humans, while rats experience a decrease in blood pressure in high-altitude environments. For example, research indicates that the Asian house rat significantly enhances hypoxia tolerance through genomic-level adaptive evolution (such as mutations in the RTN4 gene) and adjustments in metabolic pathways (like differential expression of fatty acid metabolism-related genes) (Chen et al., 2021). In addition, hypoxia may trigger different physiological adaptive mechanisms. Our findings suggest that WKY rats maintain the integrity of the ACE2-MasR axis by selectively suppressing the ACE-Ang II-AT1R axis, thereby avoiding uncontrolled blood pressure. This species-specific response may be linked to their genetic background: Asian house rats form structural protection in hypoxic environments by upregulating hypoxia-responsive genes (such as TRH and MMP12) and angiogenesis-related pathways (Chen et al., 2021). Therefore, a thorough critical evaluation of these differences is necessary to better understand the physiological responses of different species under hypoxic conditions.

This study has some limitations. Firstly, it focuses exclusively on the effects of long-term exposure to high-altitude hypoxic environments on blood pressure, without comparing different exposure durations. As a result, it is challenging to fully illustrate the dynamic changes in the relationship between hypoxia and blood pressure. Secondly, the study has not explored the interaction mechanisms among various physiological indicators during the process of blood pressure reduction in high-altitude hypoxic conditions. Thirdly, the lack of validation in other causative models limits the generalisability of our conclusions. Finally, this study did not investigate the mechanisms through which thyroid hormones affect blood pressure and failed to accurately distinguish the differences in the effect size of the RAS. These design limitations prevent a complete understanding of the multifactorial interactions involved in blood pressure regulation.

## 4 Materials and methods

### 4.1 Animals and experimental treatments

Experiments were conducted on 12, 8-week-old, male SHRs and 12 age-matched male normotensive WKY rats purchased from Huafukang Biotechnology Co. Ltd., Beijing, China (certificate number: 2019-0,008). The rats were housed per cage in separate rooms on a 12-h light/12-h dark cycle at a constant temperature of 22°C ± 2°C and humidity of 55% ± 10%, with food and water intake *ad libitum*. All animal experiments were conducted according to the stipulations outlined in the Regulations for the Administration of Affairs Concerning Experimental Animals (2017 Revision) and the Laboratory Animals: General Requirements for Animal Experiments (GB/T 35,823-2018). These experiments were also approved by the Ethics Committee of Experimental Animal Use of Qinghai University (approval number: PJ202401-74; 15 March 2024).

The animals were studied after 10 weeks of exposure to high-altitude or control conditions. SHRs were randomly divided into two groups: a high-altitude hypoxia group (SHR-H) (4,300 m; PaO_2_, 12.5 kPa) and a control group (SHR-C) (1,660 m; PaO_2_, 17.5 kPa). WKY rats were randomly divided into two groups: a high-altitude hypoxia group (WKY-H) (4,300 m; PaO_2_, 12.5 kPa) and a control group (WKY-C) (1,660 m; PaO_2_, 17.5 kPa). Each group consisted of six rats. The rats in the control groups were kept at an altitude of approximately 1,660 m in Minhe County, Qinghai Province, China. They were transported by rail from Xi’an City. The rats in the high-altitude hypoxia groups were kept at an altitude of approximately 4,300 m in Hua Shixia Town, Qinghai Province, China. They were transported by rail from Xi’an to Xining City, and then by bus to Hua Shixia Town. The entire trip took 10 h to complete.

### 4.2 Blood pressure measurements

In the resting state, SBP, DBP, MBP, and HR of the caudal artery in rats were measured using a BP-2010 A automatic non-invasive blood pressure monitor (Softron Biotechnology Co. Ltd., Beijing, China) using the tail-cuff method (Hao et al., 2014; Liu et al., 2023). All blood pressure measurements were performed in a dedicated experimental facility by a single trained experimenter. To measure blood pressure using this monitor, the device was first opened, and the air hose was connected to the pressure sensor. Next, each rat was secured within a mesh bag and placed in an insulation sleeve before being positioned in the rat holder. Finally, the pressure sensor was aligned with the base of the rat tail, ensuring that the marked tip corresponded to the tip of the rat tail (Liu et al., 2023; Krege et al., 1995). Each rat underwent a 7-day pre-adaptation protocol to acclimate to the experimental environment before measurement. Three consecutive measurements were obtained for each rat at the beginning of the experiment to determine the mean SBP, DBP, MBP, and HR.

### 4.3 Echocardiography examination

The rats were anesthetised by isoflurane inhalation. The anaesthesia machine was adjusted to deliver 1% oxygen and 4% isoflurane to induce anaesthesia, followed by maintenance of anaesthesia with 1% oxygen and 2.5% isoflurane. All experimental measurements were conducted in a dedicated experimental facility by a single trained experimenter to ensure procedural consistency. The rats were then secured to the operation platform of a small-animal ultrasound system (Vevo 3,100; Fujifilm Visualsonics Inc., Montreal, Canada) using a three-dimensional positioning fixture to maintain a supine posture at 30°, with their heads and limbs fixed and the anterior chest exposed. Preparation involved shaving the thoracic area and applying an ultrasound coupling gel to the precordial area to eliminate air interference. A specialised ultrasound probe (MS 250, 20 MHz; axial resolution 75 μm; Visual-Sonics, Toronto, Canada) was used to capture and save two-dimensional echocardiogram images in the left ventricular long- and short-axis views. Cardiac parameters, including left ventricular CO, left ventricular EF, left ventricular FS, left ventricular stroke volume, LVDd, LVSd, LVAMs, and LVAMd, were measured using M-mode over three consecutive cardiac cycles, and the average of the three values was calculated. The images were analysed using Vevo LAB software (version 3.2.6; Fujifilm Visualsonics, Toronto, Canada).

### 4.4 Measuring mPAP, RVSP, and RVEDP

After 10 weeks of exposure, the rats were anesthetised with urethane (0.5 mL/100 g, intraperitoneally). After the rats were stabilised and fixed in the supine position, the right external jugular vein was separated and a heparinised bent polyethylene catheter connected to the MP150 16-lead physiological signal acquisition system (Biopac, Hopkinton, MA) was inserted into the right ventricle and pulmonary artery through the right external jugular vein. Waveform changes were observed, and mPAP, RVSP, and RVEDP data were collected for analysis (Wang et al., 2023).

### 4.5 Histochemical staining

The heart, liver, lung, kidney, and abdominal aortic tissues of the rats in each group were fixed in 10% paraformaldehyde and embedded in paraffin. Afterwards, specimens were cut into 5-µm sections using a tissue slicer and stained with haematoxylin and eosin. Images were acquired using a Pannoramic 250 digital slice scanner (3DHISTECH, Budapest, Hungary), and the medial membrane thickness of the abdominal aortic tissues was measured using CaseViewer image analysis software (version 2.4.0; 3DHISTECH).

To observe the extracellular matrix content of abdominal aortic tissues, the slides were stained with Masson’s trichrome and Weigert’s resorcin-fuchsin to highlight collagen (blue) and elastic fibres (black-violet), respectively. The tissue areas of collagen and elastic fibres in the acquired images were measured using the Image-Pro Plus 6.0 analysis system (Media Cybernetics, Silver Spring, MD), and the percentages of collagen and elastic fibre expression were calculated.

### 4.6 Haematological and biochemical parameters

To examine haematological indicators in the rats, whole blood was collected from the abdominal aorta and placed in an anticoagulant tube containing EDTA-2Na. Routine blood examinations were performed using an XN-9000 automatic haematology analyser (Sysmex Corp., Kobe, Japan), including white blood cell and RBC count, haemoglobin and haematocrit concentration, MCV, MCH, and platelet count measurements.

Whole blood was also collected from the abdominal aorta and placed in tubes without anticoagulants and centrifuged. Blood biochemical parameters, including alanine aminotransferase, aspartate aminotransferase, creatinine, glucose, urea, LDL-C, TC, and triglycerides, were measured using an AU5800 automatic biochemistry analyser (Beckman Coulter Corp., Brea, CA).

### 4.7 Immunohistochemistry

Immunohistochemistry was used to detect the expression of Ang I, ACE, Ang II, AT1R, ACE2, Ang 1–7, and MasR in the abdominal aortic tissues. Micron-thick sections were cut from paraffin-embedded abdominal aortic tissue blocks using a microtome at room temperature. The sections were deparaffinised in xylene and hydrated using a graded series of ethanol solutions. Antigen retrieval consisted of a microwave heat treatment (high heat for 10 min, ceasefire for 8 min, and medium-high heat for 10 min) with sections in citrate buffer (pH 6.0). These sections were then incubated with primary antibodies, including rabbit anti-rat Ang I (Bs-1707R, 1:100, Bioss, Beijing, China), ACE (24743-1-AP, 1:200, Proteintech, Wuhan, China), Ang II (Bs-0587R, 1:100, Bioss), AT1R (25343-1-AP, 1:100, Proteintech), ACE2 (21115-1-AP, 1:400, Proteintech), Ang 1–7 (bs-20101R, 1:100, Bioss), and MasR (bs-5926R, 1:100, Bioss). This was followed by incubation with the HRP-conjugated secondary antibody (goat anti-rabbit, GB23303, 1:100, Servicebio, Wuhan, China). Diaminobenzidine was used at room temperature as the chromogen, and the staining time was controlled using a microscope. Images of the stained sections were obtained using a microcamera system, and the proportion of positive areas was analysed using the HALO data analysis system (IndicaLabs., Albuquerque, NM).

### 4.8 Statistical analysis

All statistical analyses were performed using Statistical Package for the Social Sciences (version 27.0; SPSS Inc., Chicago, IL, United States). Comparisons between multiple groups were performed using the Kruskal–Wallis H test, with *post hoc* comparisons made using Dunn’s method; data are presented as the medians and interquartile ranges. *P* < 0.05 was considered statistically significant. Graphs were generated using GraphPad Prism software (version 8; GraphPad Software, Inc., La Jolla, CA, United States).

## 5 Conclusion

In conclusion, long-term exposure to high-altitude hypoxia reduces blood pressure in SHRs, likely due to a combination of cardiac functional compensation failure, vascular remodelling, and the simultaneous inhibition of the ACE-Ang II-AT1R and ACE2-Ang1-7-MasR axes. However, this mechanism is species-specific and contrasts with human responses to high-altitude hypoxia, where blood pressure typically increases due to distinct regulatory pathways.

## Data Availability

The original contributions presented in the study are included in the article/supplementary material, further inquiries can be directed to the corresponding authors.
